# Primary Intracranial Cholesteatoma Presenting as a Cerebellar Mass Without Otologic Symptoms

**DOI:** 10.7759/cureus.104401

**Published:** 2026-02-27

**Authors:** Elizabeth Blanco Espinosa, Ricardo Marlon Saro Del Valle, Idania Cruzata Matos, Adrian Zelada Valdes, Siurys D Mata Rieumont, Raysa Garces Ruiz

**Affiliations:** 1 General Practice, CEDA Orthopedic Group, Miami, USA; 2 General Surgery, Hospital Universitario Arnaldo Milian Castro, Santa Clara, CUB; 3 Neurology, Hospital General Dr. Juan Bruno Zayas Alfonso, Santiago de Cuba, CUB; 4 General Medicine, HCA Healthcare, Nevada, USA; 5 Pathology, Jackson Memorial Hospital, Miami, USA; 6 Medicine, Universidad de Ciencias Médicas, Ciego de Ávila, CUB; 7 Family Medicine, Universidad de Ciencias Médicas de Camagüey, Camagüey, CUB

**Keywords:** cerebellar syndrome, diffusion-weighted imaging, epidermoid tumor, intracranial cholesteatoma, posterior fossa

## Abstract

Intracranial cholesteatoma (epidermoid tumor) is an uncommon benign lesion that may demonstrate locally aggressive behavior, resulting in bone erosion and significant mass effect. Posterior fossa involvement presenting as a cerebellar syndrome in the absence of otologic symptoms is exceptionally rare and may closely mimic primary intracranial neoplasms, posing a diagnostic challenge. We report the case of a 29-year-old male who developed progressive vertigo, gait instability, and projectile vomiting one month after an appendectomy performed under spinal anesthesia. Neurological examination revealed mixed vertical and horizontal spontaneous nystagmus, limb dysmetria, and truncal ataxia. MRI showed a large posterior fossa lesion with marked diffusion restriction, absence of contrast enhancement, and extension into the cerebellopontine angle and upper cervical canal. The patient underwent suboccipital craniectomy with microsurgical excision of the lesion. Intraoperatively, a well-circumscribed, pearly white, encapsulated lesion containing keratinous material was identified. Histopathological examination confirmed the diagnosis of cholesteatoma. Postoperatively, the patient experienced progressive resolution of neurological symptoms, and a follow-up MRI at six months demonstrated no evidence of recurrence. This case emphasizes that intracranial cholesteatoma with posterior fossa extension should be included in the differential diagnosis of cerebellar masses, even in the absence of otologic symptoms. Diffusion-weighted MRI is essential for accurate diagnosis, and early complete surgical excision is associated with an excellent prognosis.

## Introduction

Cholesteatoma is a benign yet locally destructive pathological entity characterized by the presence of keratinizing stratified squamous epithelium in anatomical locations where it is not physiologically present, most commonly within the middle ear and mastoid air cell system [1]. The progressive accumulation of keratin debris within these lesions promotes chronic inflammation and enzymatic bone resorption, which can result in the erosion of adjacent structures in cases involving the temporal bone despite the absence of malignant histopathological features [2].

Most cases correspond to acquired cholesteatomas, typically associated with chronic middle ear disease and eustachian tube dysfunction. Sustained negative pressure within the middle ear facilitates tympanic membrane retraction and epithelial migration, ultimately leading to keratin retention and progressive lesion expansion [2]. In contrast, congenital cholesteatomas represent a smaller subset and are thought to arise from embryonic epithelial remnants that may remain dormant for years before becoming clinically apparent [3].

It is important to distinguish middle-ear (aural) cholesteatomas from primary intracranial epidermoid tumors, although both share similar histological features. Middle-ear cholesteatomas originate from the tympanic membrane or middle-ear mucosa and are associated with chronic inflammation and bone erosion, whereas primary intracranial epidermoid tumors arise from ectodermal epithelial inclusions during neural tube closure between the third and fifth weeks of embryogenesis. These embryologically derived lesions develop independently of middle-ear pathology and do not require prior otologic disease. They are most commonly located in the cerebellopontine angle and parasellar region and typically present as well-circumscribed, pearly white masses.

From an epidemiological perspective, middle-ear cholesteatoma has an estimated annual incidence of 3-9 per 100,000 individuals in Western populations, with a slight male predominance [4]. In contrast, primary intracranial epidermoid tumors account for approximately 0.2-1.8% of all intracranial tumors [5], underscoring the rarity of this entity. Posterior fossa epidermoid tumors represent the majority of intracranial cases; however, extension into the cervical canal without associated otologic disease remains exceedingly uncommon and is largely described in isolated case reports.

Despite their benign classification, intracranial epidermoid tumors may produce significant morbidity due to mass effect and involvement of adjacent neurovascular structures. Clinical manifestations depend on anatomical location and may include cranial nerve deficits, cerebellar dysfunction, hydrocephalus, or brainstem compression. Unlike middle-ear cholesteatomas, bone erosion is not a defining feature of primary intracranial lesions [6].

Intracranial extension of middle-ear cholesteatoma is rare and has been reported in approximately 5-10% of advanced otologic cases in contemporary series. However, primary intracranial epidermoid tumors of the posterior fossa may present without any prior otologic history, frequently manifesting with atypical neurological symptoms rather than classic ear-related findings. Therefore, distinguishing primary intracranial epidermoid tumors from secondary otogenic intracranial extension is essential, as the pathogenesis, clinical course, and surgical management strategies differ substantially.

The present case illustrates an unusual neurological presentation of a posterior fossa cholesteatoma in the absence of otologic disease, highlighting the importance of distinguishing primary intracranial lesions from secondary otogenic extension and emphasizing the role of early recognition and timely surgical intervention in preventing irreversible neurological sequelae [2,7].

## Case presentation

History and clinical findings

A 29-year-old male with no significant past medical history presented with progressively worsening neurological symptoms. He had undergone an uncomplicated appendectomy under spinal anesthesia one month prior; however, his immediate postoperative course was unremarkable. Neurological symptoms began several days after discharge and progressively worsened over the following weeks, with the onset of gait instability, visual disturbances, and recurrent episodes of projectile vomiting. There was no history of head trauma, fever, seizures, or constitutional symptoms such as weight loss or night sweats. The patient denied otologic symptoms, including hearing loss, tinnitus, otorrhea, recurrent otitis media, or prior ear surgery. He also denied prior neurological disorders or similar episodes. There was no orthostatic component to the headache, no positional worsening suggestive of cerebrospinal fluid leakage, and no clinical or laboratory evidence of meningitis or chemical meningitis.

Neurological examination

On admission, the patient was alert, oriented, and cognitively intact. Cranial nerve examination revealed mixed vertical and horizontal nystagmus. Cranial nerve VIII function was grossly intact, with no subjective hearing impairment reported. Motor strength was preserved in all four extremities, with normal muscle tone and symmetric deep tendon reflexes. Sensory examination was intact to light touch and pinprick throughout. Cerebellar testing demonstrated marked dysmetria, predominantly involving the right upper and lower extremities. Truncal ataxia was evident, with deviation of gait toward the right side and inability to perform tandem gait. Romberg testing was not contributory due to significant truncal instability. Fundoscopic examination revealed no evidence of papilledema. No signs of meningeal irritation were observed. These findings were anatomically consistent with involvement of the right cerebellar hemisphere and vermis, correlating with the lesion later identified on MRI.

Neuroimaging findings

**Figure 1:**
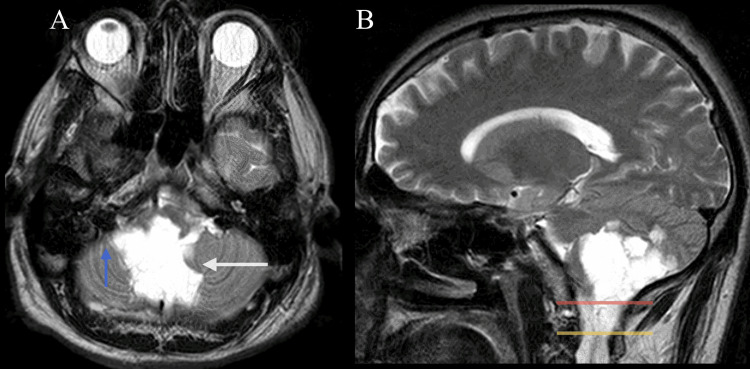
MRI of the posterior fossa lesion. A: Axial T2-weighted MRI demonstrates a large, well-circumscribed hyperintense lesion within the posterior fossa, involving the cerebellar vermis (white arrow) and right cerebellar hemisphere, with extension into the right cerebellopontine angle (blue arrow). The lesion exerts mass effect on adjacent cerebellar structures without evidence of obstructive hydrocephalus. B: Sagittal T2-weighted MRI shows inferior extension of the lesion through the foramen magnum (red line) into the upper cervical spinal canal (yellow line), with associated compression of posterior fossa structures.

Given the patient’s progressive cerebellar signs, neuroimaging was pursued. MRI was obtained and demonstrated a large, well-circumscribed lesion within the posterior fossa, involving the cerebellar vermis and right cerebellar hemisphere, measuring approximately 40 mm in its maximal axial diameter. The lesion appeared hypointense on T1-weighted sequences and hyperintense on T2-weighted images. Diffusion-weighted imaging (DWI) demonstrated marked diffusion restriction, a characteristic feature highly suggestive of an epidermoid lesion. Corresponding low apparent diffusion coefficient (ADC) values supported true diffusion restriction. No significant enhancement was observed following gadolinium administration (Figures 1A, 1B).

The lesion extended laterally into the right cerebellopontine angle and inferiorly through the foramen magnum, reaching the upper cervical spinal canal. Despite its large size and associated mass effect on adjacent cerebellar structures, no evidence of obstructive hydrocephalus was identified, and the fourth ventricle remained patent.

Although the early onset of headaches and vertigo initially raised suspicion for a post-dural puncture complication, the subsequent clinical progression and neuroimaging findings demonstrated an underlying intracranial mass unrelated to the anesthetic procedure.

**Figure 2:**
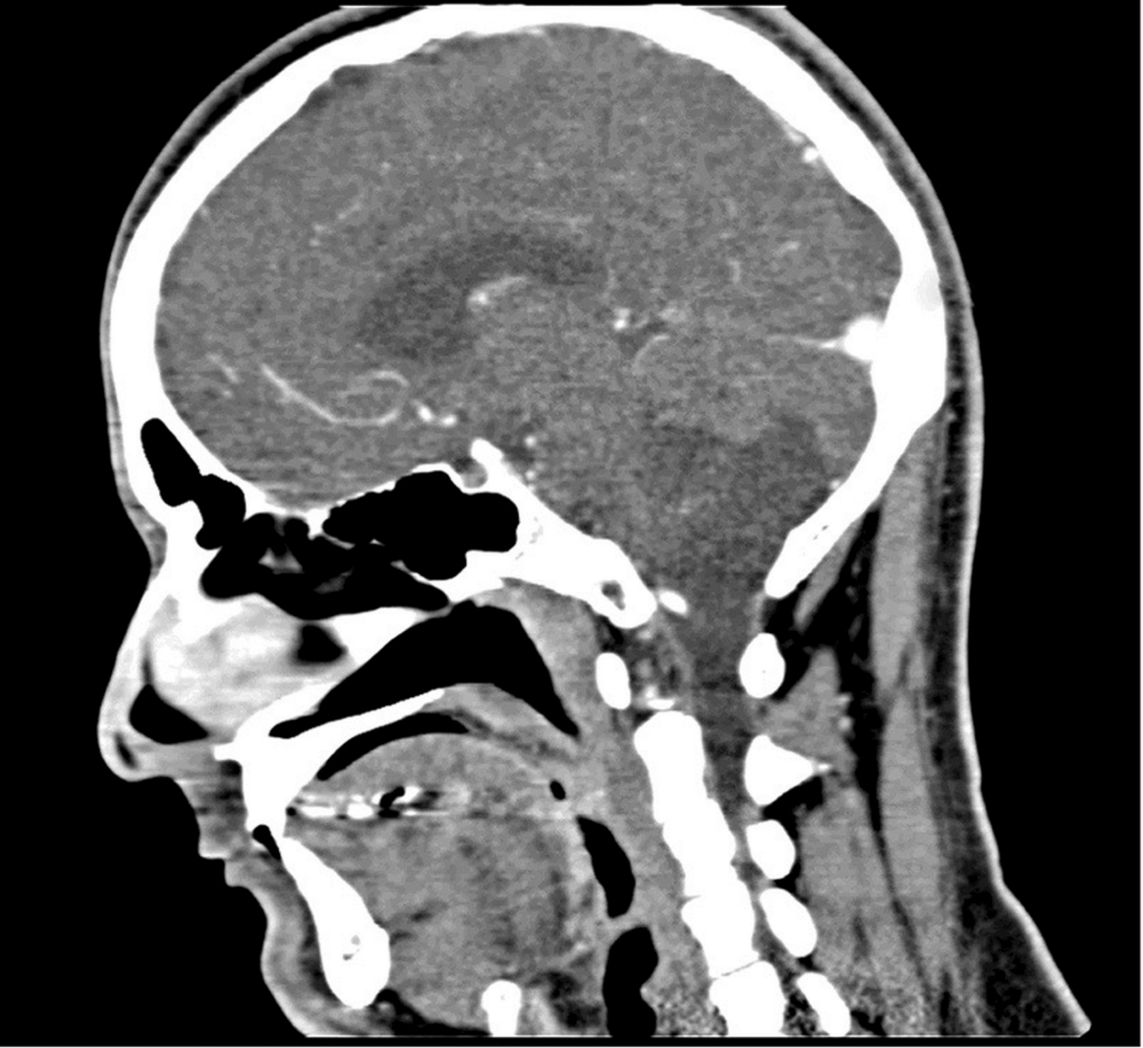
Preoperative sagittal CT scan showing a posterior fossa mass involving the cerebellar region.

A preoperative non-contrast CT scan confirmed the presence of a posterior fossa mass. The brain parenchyma showed normal gray-white matter differentiation, with no midline shift, or acute intracranial hemorrhage, and no significant mass effect; however, subtle posterior fossa compression was better appreciated on MRI due to superior soft tissue resolution. The ventricular system appeared normal in size and configuration, without signs of hydrocephalus. The skull base and calvarium were intact, with no evidence of petrous temporal bone erosion, middle-ear opacification, or communication with the mastoid air cell system, strongly supporting a primary intracranial epidermoid lesion rather than secondary otogenic extension (Figure 2).

Differential diagnosis

Based on the patient’s age, anatomical location, and radiological characteristics, the differential diagnosis included epidermoid tumor (cholesteatoma), arachnoid cyst, ependymoma, cerebellar astrocytoma, and medulloblastoma, the latter considered unlikely given the patient’s age. Hemangioblastoma was also considered but deemed less likely due to the absence of cystic components. The marked diffusion restriction on DWI with corresponding low ADC values was critical in distinguishing this lesion from an arachnoid cyst, which typically follows cerebrospinal fluid signal and does not restrict diffusion. The complete absence of otologic symptoms was a critical diagnostic element, as it reduced the likelihood of secondary intracranial extension from middle-ear cholesteatoma and supported consideration of a primary intracranial process.

Surgical management

The case was discussed in a multidisciplinary setting. Given the progressive neurological deterioration and extensive posterior fossa involvement, surgical intervention was recommended. A contrast-enhanced MRI was obtained preoperatively to further delineate lesion margins and assess its relationship with surrounding neurovascular structures. The patient subsequently underwent a suboccipital posterior fossa craniectomy with microsurgical excision of the lesion. Intraoperatively, a pearly white lesion with friable keratinous content was encountered. The capsule consisted of a thin epithelial lining densely adherent in focal areas to the brainstem and lower cranial nerves. Careful microsurgical dissection allowed near-total removal of the epithelial lining; however, in areas of firm adherence to critical neurovascular structures, a microscopic remnant was intentionally left to avoid neurological injury.

Histopathological findings

Histopathological examination demonstrated stratified squamous epithelium with abundant lamellated keratin debris and no evidence of malignancy, confirming the diagnosis of cholesteatoma (epidermoid tumor). No dermal appendages were identified, effectively excluding a dermoid cyst.

Postoperative course and follow-up

The postoperative course was favorable, with progressive resolution of vertigo, vomiting, and nystagmus. Gait instability and cerebellar dysfunction improved gradually with neurological rehabilitation. No new neurological deficits were observed. The patient was discharged in stable condition and followed on an outpatient basis. At the six-month follow-up, he remained asymptomatic with complete clinical recovery.

**Figure 3:**
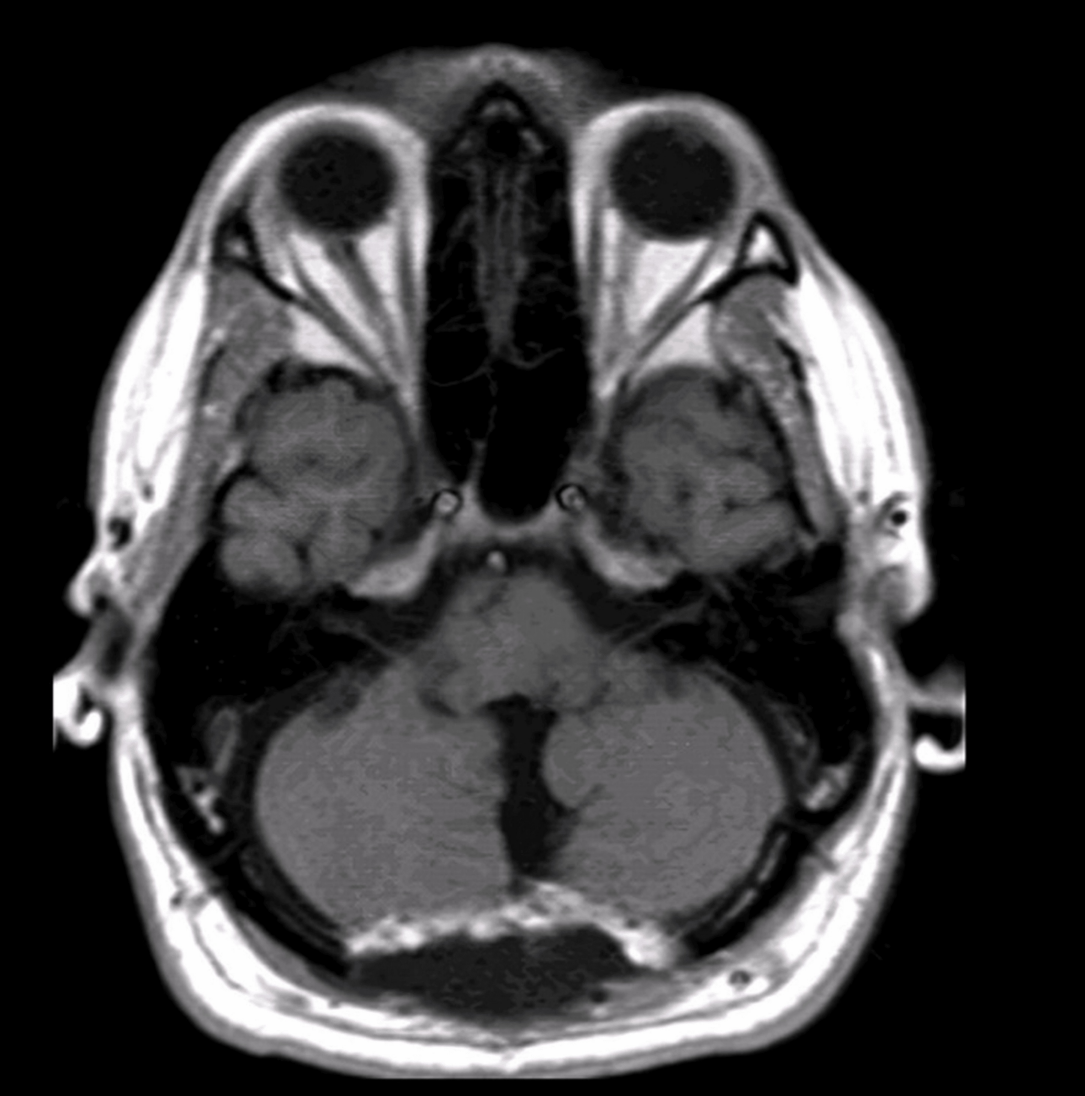
Six-month postoperative axial MRI showing postoperative changes in the posterior fossa without evidence of recurrence.

Axial MRI of the brain obtained six months after the surgical intervention demonstrated postoperative changes within the posterior fossa. There was no evidence of residual or recurrent mass lesion. The cerebellar hemispheres and vermis showed preserved morphology, with no significant mass effect on adjacent structures. The brainstem and surrounding posterior fossa structures appeared appropriately decompressed, and the fourth ventricle was normal in size and configuration, without evidence of obstructive hydrocephalus. No abnormal signal intensity suggestive of tumor recurrence was identified on this sequence (Figure 3).

**Figure 4:**
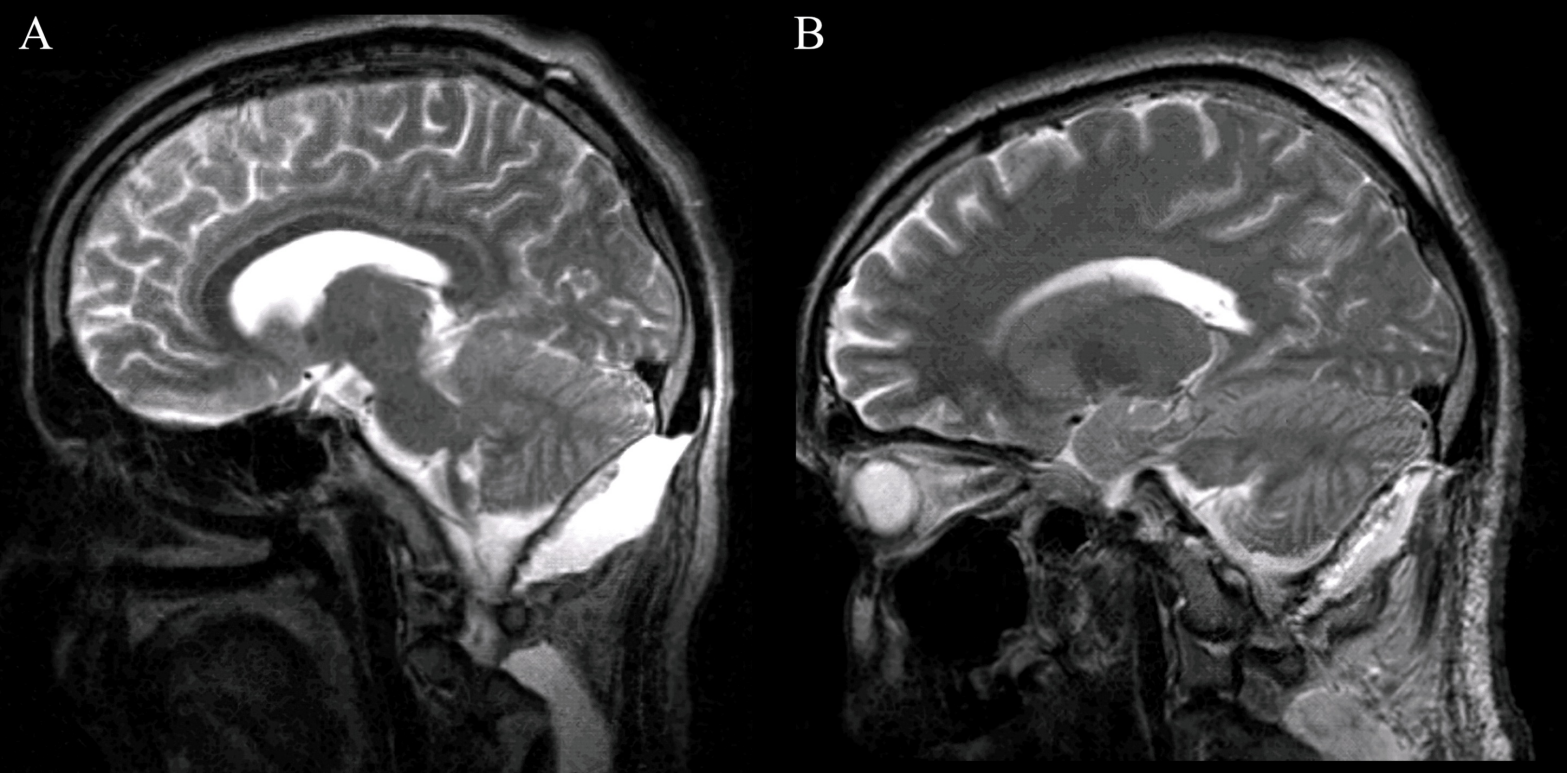
(A, B) Sagittal brain MRI obtained nine months postoperatively demonstrating postoperative changes in the posterior fossa without evidence of residual or recurrent lesion.

Sagittal T2-weighted MRI obtained nine months after the surgical intervention demonstrated postoperative changes within the posterior fossa with adequate decompression of the cerebellum and brainstem. There was no evidence of residual or recurrent lesion. The cerebellar hemispheres and vermis showed preserved morphology, and the fourth ventricle remained normal in size and configuration, without evidence of obstructive hydrocephalus. No abnormal signal intensity suggestive of tumor recurrence was identified (Figure 4).

## Discussion

Primary intracranial cholesteatoma is a rare congenital lesion that differs fundamentally from acquired middle-ear cholesteatoma in its pathogenesis and clinical behavior. Unlike cases of secondary intracranial extension from middle-ear cholesteatoma, which occur through progressive temporal bone erosion, the present lesion demonstrated no radiological evidence of petrous bone destruction or communication with the middle ear. This strongly supports a primary intracranial epidermoid cyst rather than otogenic spread [6,7]. While most reported posterior fossa epidermoid tumors arise in the cerebellopontine angle and present with cranial nerve deficits, cases with predominant cerebellar syndrome and cervical canal extension without hydrocephalus remain exceptionally rare.

Intracranial cholesteatoma has been reported in a limited number of anatomical locations and almost exclusively as isolated case reports or small case series. The reported intracranial locations and approximate case counts are summarized in Table 1. Importantly, the cases summarized include exclusively primary intracranial lesions, deliberately excluding secondary otogenic extensions to avoid data mixing and ensure conceptual clarity.

**Table 1:** Reported intracranial cholesteatoma locations and approximate number of published cases. Case counts are approximate and derived from a cumulative review of published case reports and small case series. Reports classified exclusively as epidermoid tumors were excluded unless explicitly described as cholesteatoma.

Intracranial structure involved	Approximate number of reported cases
Cerebellopontine angle/Posterior fossa	~50–70
Petrous apex/Skull base	~30–45
Temporal lobe (intraparenchymal)	~15–25
Intraventricular (lateral/third/fourth ventricle)	~8–12
Intradiploic (calvarial with intracranial extension)	~15–20
Sellar/Suprasellar region	~5–7
Frontal or parietal lobes	~5–8
Thalamus/Deep gray nuclei	1–2
Diffuse intraparenchymal (non-temporal)	<5

Recent literature consistently describes cholesteatoma as a condition capable of producing severe intracranial and extracranial complications despite its benign histopathological nature. Contemporary reviews report sequelae such as meningitis, epidural and subdural empyema, cerebellar abscess, venous sinus thrombosis, cerebrospinal fluid leakage, and ventriculitis, most commonly resulting from chronic bone erosion and secondary infection. Large institutional series further confirm that venous sinus involvement and suppurative intracranial complications may constitute the initial presentation of previously undiagnosed disease (Table 2) [8-15].

**Table 2:** Intracranial complications and clinical manifestations associated with cholesteatoma. Complications summarized from published case reports and retrospective series.

Category	Reported manifestations
Infectious complications	Meningitis; epidural empyema; subdural empyema; brain abscess; cerebellar abscess; ventriculitis
Vascular complications	Venous sinus thrombosis
Cerebrospinal fluid-related complications	Cerebrospinal fluid leakage
Neurological manifestations	Focal neurological deficits; cerebellar signs; altered mental status
Extracranial extensions	Deep neck space infection; combined extracranial–intracranial abscess
Non-infectious mass effect	Space-occupying lesions with neurological compromise (rare)

The absence of infectious or inflammatory features in the present patient further supports a primary intracranial origin rather than an otogenic process. Although infectious complications were absent, the literature illustrates the broad spectrum of cerebellar involvement associated with cholesteatoma, ranging from localized mass effect to life-threatening intracranial pathology. In contrast, a predominantly non-infectious space-occupying lesion producing significant mass effect without acute inflammatory signs, as observed in this case, is distinctly uncommon and may closely mimic primary intracranial neoplasms, thereby complicating the diagnostic process and potentially delaying definitive management.

The neurological presentation in this case, i.e., progressive cerebellar signs and positional instability without obstructive hydrocephalus, reflects the slow-growing nature of these lesions. This behavior has been noted in other rare intracranial cholesteatoma reports, where patients present insidiously over years with non-specific symptoms such as headache or imbalance and may lack classic otologic signs such as otorrhea or hearing loss [7-9,14,15]. This silent progression underscores the importance of considering cholesteatoma in the differential diagnosis even in the absence of overt ear disease.

Accurate radiological differentiation is essential in the evaluation of suspected cholesteatoma, particularly in atypical intracranial presentations. In our case, the lesion measured approximately 40 mm in maximal diameter on T2-weighted sequences, producing significant compression of adjacent cerebellar structures and caudal extension into the upper cervical canal without ventricular obstruction. DWI demonstrates high sensitivity for cholesteatoma by revealing characteristic restricted diffusion related to keratin content, thereby facilitating reliable differentiation from arachnoid cysts and cystic or solid neoplasms [16].

Kumar described the technical principles and diagnostic performance of diffusion-weighted MRI, elucidating the biophysical basis of diffusion restriction in keratinizing lesions [16]. Diffusion-weighted imaging has been widely used in the evaluation of epidermoid and related keratinizing lesions, contributing to lesion characterization based on diffusion signal properties. Although quantitative ADC values were not systematically recorded in this case, qualitative diffusion restriction was observed, consistent with commonly described imaging features of epidermoid lesions.

In complex posterior fossa lesions lacking typical clinical features, diffusion-weighted imaging often proves indispensable for establishing an accurate diagnosis and guiding timely surgical management.

Surgical management remains the cornerstone of treatment for intracranial cholesteatoma. When feasible, complete resection is preferred, as it effectively alleviates mass effect and reduces recurrence risk; however, aggressive attempts at capsule removal in cases of dense adherence to the brainstem or cranial nerves may increase the likelihood of permanent neurological deficits. The diagnostic complexity of lesions in this region has been highlighted by Bray and Sappington, who emphasized the overlapping clinical and radiological features among neoplastic, congenital, and cystic entities and the importance of advanced imaging in surgical planning [17].

In the present case, near-total resection was achieved. Although the majority of the lesion and epithelial lining were removed, a microscopic remnant was intentionally left in areas of firm adherence to the brainstem and lower cranial nerves to minimize the risk of neurological injury. Postoperative MRI demonstrated no significant residual mass effect and no evident diffusion-restricting component suggestive of substantial residual disease. The favorable postoperative course, with resolution of cerebellar signs at six-month follow-up, illustrates the potential for meaningful neurological recovery following timely and carefully balanced surgical intervention. Similarly, Costea et al. reported successful microsurgical management of a fourth ventricle epidermoid cyst, demonstrating that meticulous technique can achieve effective decompression while preserving critical neurovascular structures and yielding excellent functional outcomes [18].

Long-term outcome data provided by Omer et al. reinforce the prognostic importance of maximal safe resection and sustained imaging surveillance [19]. Given the known risk of delayed recurrence after subtotal or near-total resection of epidermoid tumors, continued long-term MRI surveillance remains essential in this case.

When compared with previously reported posterior fossa epidermoid cases, most described lesions presented either with cranial nerve deficits related to cerebellopontine angle involvement or with signs of hydrocephalus due to fourth ventricular obstruction. In contrast, the present case demonstrated extensive posterior fossa involvement with caudal cervical extension and significant mass effect in the absence of hydrocephalus or otologic symptoms. To our knowledge, reports combining posterior fossa extension into the upper cervical canal without associated ear disease or obstructive hydrocephalus remain exceedingly limited in the contemporary literature.

The uniqueness of this case lies in the absence of otologic symptoms, extensive posterior fossa involvement with caudal extension into the upper cervical canal, lack of hydrocephalus despite significant mass effect, and radiological diagnosis primarily guided by DWI. Such a constellation of findings is rarely described in the literature and may closely mimic primary cerebellar neoplasms. This case underscores the importance of maintaining a broad differential diagnosis for posterior fossa masses exhibiting diffusion restriction and highlights the critical role of comprehensive neuroimaging and timely, balanced surgical intervention in achieving favorable neurological outcomes.

## Conclusions

Primary intracranial cholesteatoma is a rare congenital lesion that may present exclusively with progressive cerebellar dysfunction in the absence of otologic symptoms. Unlike secondary otogenic cholesteatoma, primary intracranial lesions may occur without radiological evidence of temporal bone erosion or middle-ear involvement, yet still produce significant posterior fossa mass effect. Extensive posterior fossa involvement, even with caudal extension into the upper cervical canal, may occur without hydrocephalus and can closely mimic primary cerebellar neoplasms, thereby complicating diagnosis. DWI plays a pivotal role in distinguishing these lesions from arachnoid cysts and other cystic or neoplastic posterior fossa masses. Microsurgical resection with preservation of critical neurovascular structures remains the treatment of choice. Although complete epithelial removal may be technically challenging due to adherence to adjacent cranial nerves and brainstem structures, careful surgical technique can achieve excellent neurological recovery. Given the potential for microscopic residual epithelial remnants, long-term radiological follow-up remains essential, even in cases with apparent complete resection. This case contributes to the existing literature by illustrating a purely neurological presentation of primary intracranial cholesteatoma with cervical canal extension and absence of hydrocephalus, reinforcing the importance of maintaining a broad differential diagnosis in posterior fossa lesions exhibiting diffusion restriction.
