# Correction: Primary Intracranial Cholesteatoma Presenting as a Cerebellar Mass Without Otologic Symptoms

**DOI:** 10.7759/cureus.c429

**Published:** 2026-05-08

**Authors:** Elizabeth Blanco Espinosa, Ricardo Marlon Saro Del Valle, Idania Cruzata Matos, Adrian Zelada Valdes, Siurys D Mata Rieumont, Raysa Garces Ruiz

**Affiliations:** 1 General Practice, CEDA Orthopedic Group, Miami, USA; 2 General Surgery, Hospital Universitario Arnaldo Milian Castro, Santa Clara, CUB; 3 Neurology, Hospital General Dr. Juan Bruno Zayas Alfonso, Santiago de Cuba, CUB; 4 General Medicine, HCA Healthcare, Nevada, USA; 5 Pathology, Jackson Memorial Hospital, Miami, USA; 6 Medicine, Universidad de Ciencias Médicas, Ciego de Ávila, CUB; 7 Family Medicine, Universidad de Ciencias Médicas de Camagüey, Camagüey, CUB

This article has been corrected to revise the seventh paragraph in the discussion section and remove the citation for reference 17 due to its misalignment with the article conclusions. Subsequent references and citations have been renumbered accordingly.

- **Original text: **Complementing these observations, Gonçalves et al. demonstrated consistent diffusion restriction and characteristic ADC patterns across epidermoid lesions and cholesteatomas, quantitatively validating DWI as a discriminative imaging tool [17]. Although precise numerical ADC values were not systematically recorded in this case, qualitative diffusion restriction was clearly demonstrated and was consistent with previously reported epidermoid imaging characteristics.
- **Corrected text: **Diffusion-weighted imaging has been widely used in the evaluation of epidermoid and related keratinizing lesions, contributing to lesion characterization based on diffusion signal properties. Although quantitative ADC values were not systematically recorded in this case, qualitative diffusion restriction was observed, consistent with commonly described imaging features of epidermoid lesions.

